# A Patent Review on the Therapeutic Application of Monoclonal Antibodies in COVID-19

**DOI:** 10.3390/ijms222111953

**Published:** 2021-11-04

**Authors:** Syed Mohammed Basheeruddin Asdaq, Syed Imam Rabbani, Meshary Alkahtani, Meshal Meshary Aldohyan, Abdullah Mohammad Alabdulsalam, Majed Sadun Alshammari, Saleh Ahmad Alajlan, Aljawharah Binrokan, Yahya Mohzari, Ahmed Alrashed, Mohammed Kanan Alshammari, Mohd. Imran, Naira Nayeem

**Affiliations:** 1 Department of Pharmacy Practice, College of Pharmacy, AlMaarefa University, Dariyah, Riyadh 13713, Saudi Arabia; 2 Department of Pharmacology and Toxicology, College of Pharmacy, Qassim University, Buraydah 51452, Saudi Arabia; syedrabbani09@yahoo.com; 3 Department of Pharmacy, King Abdulaziz Medical City, National Guard, Riyadh 12629, Saudi Arabia; Mashary78@gmail.com (M.A.); mmd.444@hotmail.com (M.M.A.); Ph.Abdullah1989@hotmail.com (A.M.A.); majedalhosaini@gmail.com (M.S.A.); 4 Department of Pediatric Dentistry, King Fahad Medical City, Riyadh 12231, Saudi Arabia; mhospital1920@gmail.com; 5 Pharmaceutical Service Department, Children Hospital, King Fahad Medical City, Riyadh 12231, Saudi Arabia; researcher1822@gmail.com; 6 Clinical Pharmacy Department, King Saud Medical City, Riyadh 12746, Saudi Arabia; Yali2016@hotmail.com; 7 Pharmaceutical Services Administration, Inpatient Department, Main Hospital, King Fahad Medical City, Riyadh 12231, Saudi Arabia; emadasdaq@gmail.com; 8 Department of Pharmaceutical Care, Rafha Central Hospital, Rafha 76321, Saudi Arabia; ii_kanan101@outlook.com; 9 Department of Pharmaceutical Chemistry, Faculty of Pharmacy, Northern Border University, Rafha 91911, Saudi Arabia; imran.pchem@gmail.com (M.I.); naira_64@yahoo.co.in (N.N.)

**Keywords:** monoclonal antibodies, innovation, patent reviews, COVID-19, therapeutic application

## Abstract

Severe acute respiratory syndrome coronavirus 2 (SARS-CoV-2) contains spike proteins that assist the virus in entering host cells. In the absence of a specific intervention, efforts are afoot throughout the world to find an effective treatment for SARS-CoV-2. Through innovative techniques, monoclonal antibodies (MAbs) are being designed and developed to block a particular pathway of SARS-CoV-2 infection. More than 100 patent applications describing the development of MAbs and their application against SARS-CoV-2 have been registered. Most of them target the receptor binding protein so that the interaction between virus and host cell can be prevented. A few monoclonal antibodies are also being patented for the diagnosis of SARS-CoV-2. Some of them, like Regeneron^®^ have already received emergency use authorization. These protein molecules are currently preferred for high-risk patients such as those over 65 years old with compromised immunity and those with metabolic disorders such as obesity. Being highly specific in action, monoclonal antibodies offer one of the most appropriate interventions for both the prevention and treatment of SARS-CoV-2. Technological advancement has helped in producing highly efficacious MAbs. However, these agents are known to induce immunogenic and non-immunogenic reactions. More research and testing are required to establish the suitability of administering MAbs to all patients at risk of developing a severe illness. This patent study is focused on MAbs as a therapeutic option for treating COVID-19, as well as their invention, patenting information, and key characteristics.

## 1. Introduction

**Figure 1:**
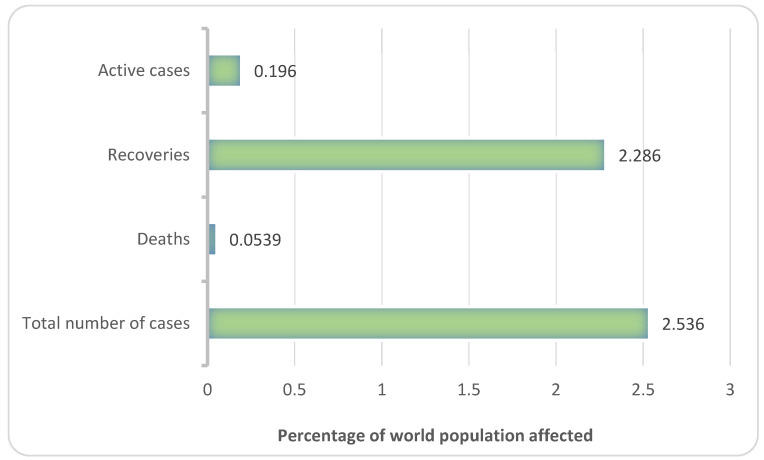
Prevalence of COVID-19 in the population (Source: https://covid19.who.int/ accessed on 10 September 2021). The figure indicates the total number of COVID-19 infections worldwide with the percentage of active cases, recoveries and deaths recorded in the population).

The novel coronavirus detected in Wuhan province of China in 2019 is called COVID-19 (coronavirus disease 2019). The infection rapidly spread to different parts of the world and was declared a pandemic by the World Health Organization (WHO) in March 2020 [1]. The prevalence of the disease in the whole population of the world as of August 2021 is represented in Figure 1.

Until now, the disease has infected millions of people and killed a significant number of them. The virus belongs to the large family of beta-coronavirus and is named as Severe Acute Respiratory Syndrome Coronavirus two (SARS-CoV-2). Other important members of this class of virus are the Middle East Respiratory Syndrome Coronavirus (MERS-CoV) and SARS-CoV-1 [2]. The virus is being mutated at regular intervals, and some of the variants have been found to be more virulent and resistant to certain vaccines. Major parts of the world are still suffering from the spread of the infection and are causing an unrepairable severe economic slowdown due to repeated lockdowns [3].

In over 80% of patients, the disease is characterized by mild symptoms, such as cough, fever, and difficult breathing. However, in aged people, immunocompromised and co-morbid patients, the infection can cause severe pneumonia, pulmonary edema, acute respiratory syndrome, sepsis, multiorgan failure and death [4]. Symptomatic diagnosis of COVID-19 is difficult and is considered inaccurate due to the resemblance to a common seasonal viral infection. Suspected individuals should be diagnosed with real-time polymerase chain reaction (RT-PCR) by collecting samples from nasal and/or throat swabs to confirm the infection [5].

SARS-CoV-2 is a beta-coronavirus containing RNA as the nuclear component. The genetic sequencing indicated that the virus has 80% similarity with SARS-CoV-1 and 96% with bat coronavirus. The outer surface of the virus contains three major components: spike (S) glycoproteins, envelope (E) and film (M) protein. The S protein binds to angiotensin-converting enzyme-2 (ACE2) located on the surface of host cells and initiates the process of infection [6]. The S protein was identified to contain two functional subunits that assist in the interaction with the host cell. The S_1_ subunits contain four core domains named S_1A_, S_1B_, S1C, and S1D, which are responsible for attaching the virus to the host. The S_2_ subunit then assists in fusion of the virus with the cellular membrane of host cells [7].

Researchers throughout the world are working overtime to find a specific medical intervention for COVID-19. Unfortunately, the studies have not yet reached the logical end in identifying a safe and effective treatment against COVID-19. The available therapeutic options to treat COVID-19 are mostly patient-specific and depend on the severity of the condition. Oxygen supplementation, dexamethasone (corticosteroids), warfarin (anticoagulant) and convalescent plasma therapy (antibody supplement) are routinely practiced [8]. The list of important anti-COVID-19 drugs in the pipeline is represented in Table 1. WHO has appealed to all countries to vaccinate their population at the earliest opportunity to prevent the spread of the disease. However, the search for a vaccine that shows a significant level of protection against all the SARS-CoV-2 variants is still under study [9].

Monoclonal antibodies (MAbs) are one of the emerging therapeutic agents efficacious for treating infectious diseases such as COVID-19. They are one of the fastest-growing pharmaceuticals and are considered to be highly specific in their action [10]. MAbs are lab-grown antibodies that specifically target the pathogen, causing its destruction immediately. Normally, MAbs are produced by B cells in patients after several days of infection. They can be isolated from recovered individuals, can be generated in the laboratory by immunizing animals and can also be constructed by molecular engineering in the laboratory [11]. Modern technology helps in the identification of specific antibodies after their production, isolation, characterization, and growth in laboratory conditions. MAbs are gaining popularity among both physicians and patients because these agents easily meet the three important requirements for being a drug: safety, efficacy and quality [12]. The MAbs currently tested against COVID-19 can either neutralize the virus action or decrease the inflammatory process due to infection. Some of them, such as bamlanivimab, sarilumab and siltuximab, have received emergency use authorization. Side effects, unpredictable bioavailability and the emergence of resistant strains are the important limitations of MAbs tested against COVID-19 [13]. The present patent review focuses on MAbs as a therapeutic option for treating COVID-19 and the innovation, patenting information and important characteristics of MAbs.

## 2. Methods

The patent review on MAbs was conducted using internet search engines, such as PubMed, Google Scholar, Science Direct and WIPO (The World Intellectual Property Organization) websites by using key words such as ‘Monoclonal’, ‘Antibodies’, ‘COVID-19’, ‘Patent Information’, ‘Clinical Trials’, ‘Mechanism’ and ‘Adverse Reactions’ [14]. The review included clinical trials conducted from the beginning of 2020, coinciding with reports of the identification of the SARS-CoV-2 genome, until the end of July 2021. The search resulted in more than 1500 total articles. However, only 88 articles were selected for the present study based on the inclusion criteria. The authors independently reviewed the titles, abstracts, and text of the articles. The information, such as English language, study center, number of subjects, study design, study protocol, dose, duration, route of administration, ethical approval, statistical methods, and biochemical estimations, were considered critical parameters for evaluating the content and were considered the inclusion criteria [15]. Only those articles containing the required information were selected for the analysis. The patent information retrieved from the WIPO is categorized separately in a table given in discussion section.

## 3. Description of Monoclonal Antibodies

Antibodies are glycoproteins formed by B cells and can be found in blood and lymph. These protein molecules are unique in structure and function in the detection, location, inactivation, and elimination of pathogens. In 1975, Kohler and Milstein demonstrated a new technology to isolate pure antibodies [16]. Technological advancement has contributed to more than 600 therapeutically used MAbs. Although most of them are targeted for cancer therapy, significant newer MAbs are being tested and approved for treating other diseases [17]. The first MAb approved by the FDA was in 1986. The drug “Muromonab-CD3” functions as an immunosuppressant and was indicated for the treatment of acute transplant rejection [11].

The concept of the use of MAb’s is based on protein therapeutics. In comparison with other low-molecular weight proteins, MAbs have high precision in producing their action and also have a longer half-life [10]. Modern molecular technologies have further tuned the specific therapeutic action of MAbs, minimized the immunogenicity and improved the risk/benefit ratio. This could be one of the reasons for MAbs achieving a better approval rate (20%) compared to other newer chemical entities (5%) [18].

Multifunctional immunoglobulins/antibodies are considered multifunctional since they show numerous cellular and humoral reactions to antigens. They are produced by the immune system and are usually polyclonal, i.e., produced by different B lymphocytes. In terms of antigen binding capacity, these antibodies behave slightly differently from one another [19]. Technological innovations have made it possible to identify one single B cell that can be stimulated to produce one specific type of antibody called a monoclonal antibody. Therefore, MAbs are homogenous preparations of antibodies obtained from single B cells and have an identical protein sequence. These antibodies possess a common antigen recognition site, affinity, biological interaction, and similar physiological effects [20].

Antibody selection is the vital step that determines the safety and efficacy of the test. Antibodies have multiple binding regions that are essential not only for antigens, but also for other cellular components. Selecting the specific region needed for antigen interaction and tailoring antibodies for this, can be achieved through a molecular engineering process [18,21]. The following sections briefly describe the techniques of production and information about MAbs used for COVID-19.

### 3.1. Vaccination of Animals

In this method, laboratory animals such as mice/rats are treated with the specific antigen. The response of the host immune system is monitored. Once seroconversion occurs, the spleen of the animals is extracted and the B cells are screened. A specific B cell is harvested to produce the MAbs. The first mAb ‘muromonab-CD3’ was obtained by this method [22].

The major concern reported with MAbs produced by this technique are allergic reactions, uncertainty in their bioavailability, and the development of human anti-mouse antibodies [23]. Approaches to minimize these drawbacks resulted in the creation of humanized or chimeric antibodies. These molecules contain the human immunoglobulin loci instead of endogenous mouse sequences and can be modified by recombinant DNA technology. In principle, the more similar MAbs are to human sequences, the less likely it is that they will induce an immunogenic response from host cells [24].

However, not all sequences can be substituted with human nucleotides because some of them are essential for the actions of MAbs. Substitution is mostly done for the critical parts of the complementarity-determining regions of MAbs [19]. Overall, the percentage of similarity for chimeric and humanized antibodies was found to be >65% and >95%, respectively. The antibodies produced by this technique lacked the complex heavy chain sequence of the mouse, which is known to be responsible for immunogenicity. Two MAbs called casirivimab and imdevimab are synthesized by using this technology for SARS-CoV-2. The MAbs target the non-overlapping epitopes of the receptor binding domain (RBD) of the S protein [25].

### 3.2. Recovering Patients

This technique is commonly used in cancer chemotherapy. The antibodies formed in the patients and having the capacity to infiltrate the tumor are identified. They are isolated from the regional lymph node and the tissues can be harvested to produce specific antibodies [24]. Peripheral blood, bone marrow and lymphoid tissues can also be used for the extraction of antibodies. MAbs isolated from this technique were found to be useful in the treatment of HIV. Bamlanivimab is a new MAb that has been tested against SARS-CoV-2 infection and is synthesized by isolating from recovered patients [25].

### 3.3. Screening of the Antibody Library

MAbs are constructed by molecular engineering in the laboratory and are selected based on their binding properties to the specific antigen. Such large, diverse Mabs were frequently produced using phage display and/or combinational techniques [26]. In vitro screening methods generally identify specific MAbs from large and diverse libraries. The size of the MAbs can be manipulated depending on the investigators’ requirements. Adalimumab, Raxibacumab and Belimumab are the examples of MAbs obtained by this method and are being tested against COVID-19 [25,27]. A brief summary of the MAbs developed through different mechanisms is represented in Table 2.

### 3.4. Mass Production

The hybridoma technique is usually adopted for the mass production of MAbs for therapeutic use. The hybridoma cells are obtained by fusing the antibody-producing cell with a myeloma B cell. Myeloma B cells are also called partner cells. These cells must be non-proliferative, otherwise, they will start producing their own antibodies [30]. Immortalization screening is used to assess this and is done by allowing the hybridoma cells to grow in a specialized growth medium. The ingredients of the media mostly support the growth of antibody-producing cells. The presence of aminopterin in the culture media distinguishes the properly fused hybridoma cells and only supports the growth of these cells [25]. The synthesized antibodies are then screened by immunoassay techniques by determining the binding characteristics of the target antigens. Other common techniques utilized for immortalization of COVID-19 MAbs are transfection with an immortalizing virus or by using Chinese hamster ovary cell lines that act as immortal cell culture lines [31].

### 3.5. MAb Modification

Clinically used MAbs require several modifications in terms of their molecular weight, glycosylation, and disulfide bond formation. Carefully selected eukaryotic production systems are used for these post-production modifications of MAbs. Yeast is also being considered for mass production of therapeutically important MAbs since they have a faster multiplying capacity [32]. Innovations have also been made to use antibody fragments instead of full-length antibodies. The fragments retain the essential binding sites for the antigen and are reported to enhance the pharmacokinetic characteristics and efficacy by penetrating inside cells/tissues [33]. Popular molecular biological methods such as phage display are used to create fragments of antibodies and a few such examples include fragment antigen binding (Fab), single-chain variable fragment (ScFv) and single-domain antibody (SdAb) [34].

### 3.6. Mechanism of Action

The overview of the mechanism of action of MAbs depends on their interaction with the target antigen. Being a biological substance, two important characteristics of MAbs, such as their binding property and ability to recruit other immune cells needed to kill the target cells, determine their safety and efficacy [35]. The binding characteristic depends on the complementarity-determining region of MAbs. The Fc (fragment crystallizable) portion of MAbs is responsible for the requirements of other immune cells. The Fc portion binding to target cells activates a cascade of complementary events resulting in the killing/destruction of the antigen [30]. Different mechanisms follow for this lethal action and include antibody-dependent cellular toxicity, antibody-mediated phagocytosis by monocytes/macrophages, or complement-dependent cytotoxicity. In some cases, MAbs are used to deliver toxic substances to target sites to produce the killing action [36].

Fc receptors are found on different types of cells, such as lymphocytes, neutrophils, monocytes, dendritic cells, and epithelial cells. The Fc portion of MAbs can be engineered to activate specific types of immune cells [32]. The glycosylation status of the Fc portion of MAbs is important for altering the effector function and determining the half-life of the molecule. The action of MAbs against infection causing agents is reported to be the same as natural humoral immunity. They tend to attack a specific component (such as RBD of SARS-CoV-2) of the microbe, thus interfering in the process of pathogenesis [37].

### 3.7. MAb Pharmacokinetics

The main objective for selecting a route of administration is to achieve a therapeutic concentration in the plasma. MAbs are protein molecules, hence they are required to be administered by parenteral routes, such as intravenous or subcutaneous. The oral route of administration is also possible for certain intestinal indications. Once in the circulation, MAbs are distributed in the different body compartments by hydrostatic and osmotic pressure [32]. The half-life of MAbs is determined by their binding characteristics with the target tissues; some have been reported to last a few hours, while others have been reported to stay in the body for days. The dosage of MAbs is usually determined depending on the body weight and body mass. Most MAbs are stable in the systemic circulation and do not undergo degradation. Elimination of MAbs occurs through reticuloendothelial macrophages. Typically, MAbs do not depend on the liver cytochrome P450 enzyme system, and the chances of a drug–drug interaction are limited with their administration [38].

### 3.8. Adverse Effects of MAbs

MAbs are used clinically under special circumstances. Their use in practice requires strict medical supervision by an expert. Since they are biological products, administration carries the risk of an immunological reaction. This needs to be explained to the patient before the therapy begins and practicing centers must have the facilities to manage them. Some of the common reactions associated with MAbs treatment include infusion reactions (irritation at the site of injection, increase in body temperature, pruritus, rarely life-threatening anaphylaxis), dermatological, endocrine, gastrointestinal, and other inflammatory reactions [39].

In addition to these, cytokine release syndrome is one more important immunological reaction associated with MAbs therapy. This reaction occurs due to the sudden release of inflammatory cytokines by T lymphocytes, resulting in fever, headache, nausea, malaise, hypotension, rash, chills, dyspnea, and tachycardia [40]. Drug resistance has also been reported after the administration of MAbs in certain cancer patients. Anti-mAb antibodies have been identified as the cause of drug resistance [30].

### 3.9. Prospects for MAbs as Preferred Therapeutic Agents in the Future

Research on MAbs has undergone tremendous development in the last 25 years. Several MAbs were identified and found to be useful in areas such as biochemistry, molecular/cellular biology, medical research, and gene therapy. Technological advancement in the basic biological sciences has enhanced the recognition of the host cell action by pathogens and their toxins, the reaction of body defense cells to transplanted organs, and pathways of carcinogenesis and immunological disorders [41].

A significant improvement in the efficacy and safety of these classes of drugs took place after identifying these molecular mechanisms of diseases. Further understanding of the mechanism of host cell immunogenicity has increased the avenues for using MAbs for other diseases [42]. In addition, multitargeted MAbs are used to treat diseases affecting different organs of the body. Currently, the therapeutic indications of MAbs cover a wide range of diseases, such as asthma, arthritis, psoriasis, Crohn’s disease, transplant rejection, ulcerative colitis, uveitis, spondylitis, migraine headaches, and infectious diseases [43].

In this way, these agents were specifically targeted to treat the disease. Anti-CFRP receptor antibodies (Erenumab), anti-fibroblast growth factor 23 (FGF 23) antibodies (Burosumab), and anti-Willebrand factor antibodies (Caplacizumab) are some of the important MAbs that have revolutionized the approach to treating disease [28,44,45]. Research is also in progress to isolate human antibodies from patients who have recovered from Middle Eastern Respiratory Syndrome Coronavirus (MERS-CoV). Attempts have been made to grow these antibodies in the lab [46]. Concurrent use of MAbs with other therapeutic agents, such as chemotherapy, radiotherapy, hormonal replacement, and other biological agents is also being tested, and the results suggest that such a combination has the potential to be an effective treatment [28]. The conjugation of antibodies with other therapeutic agents with the help of advanced technology is reported to provide novelty in the management of diseases. Some of the conjugates being tested include immuno-cytokines, antibody-drug conjugates, antibody-radionuclide conjugates, bispecific antibodies, immunoliposomes and chimeric antigen receptor T cell therapy [28,47].

Bifunctional/bispecific antibodies are another concept where two immunoglobulin chains are fused into a single molecule. The MAbs bind to two different antigens and bring them together in close physical proximity, producing a new biological function. Emicizumab is an example that brings factor X and factor VIII together to initiate the coagulation cascade used in the prophylaxis of bleeding disorders such as hemophilia A [48].

MAbs has also been used to deliver a drug substance to a specific site in the body. The bonding between the drug and MAbs is done carefully in such a manner that they do not undergo dissociation/degradation before reaching the target cells. One such example is Moxetumomab pasudotox. This is used to treat hairy cell leukemia [49].

Antigenization is a newer approach for delivering a vaccine molecule with the help of MAbs. The specific sequence/fragment of the antigen can be incorporated into one of the several binding domains of MAbs [50]. The MAbs, being specific in their target, deliver the vaccine molecule inside the cell. The vaccine molecule will then activate the cells to produce immunogenic antigens, leading to the production of antibodies. However, this technology is still in the preclinical stage, where bovine herpes virus B cell epitopes have been successfully grafted onto a bovine immunoglobulin molecule [51].

## 4. Patent Search

A patent literature review revealed more than 100 different MAbs for SARS-CoV-2 registered by pharmaceutical companies. The technique to produce these MAbs has been patented. We selected 88 such patents based on their content and these were reviewed and analyzed for patent status, technological innovations, research conducted, mechanism of action, side effects, contraindications/precautions, and any special description about the agents. According to the patent analysis, approximately 30% of published patents are registered by US-based companies, with the remainder registered by Chinese (10%) and UK (9%) companies [52]. Patent information about the articles retrieved from search engines, such as Google Scholar, Pubmed and Science Direct are represented in Table 3, while those retrieved from WIPO are mentioned in Table 4. WIPO is an intergovernmental organization that basically protects the intellectual property rights of the signatory bodies and functions as per the international treaties [53]. The data collected from this website is separately indicated in Table 4.

## 5. Discussion

Antibodies are produced by the immune system in response to infection. Monoclonal antibodies are developed in the laboratory and are designed to mimic and enhance the natural process of immunity. These agents are increasingly being tested and used against cancer and various types of infection [10]. Monoclonal antibodies are intended to target a particular infection process, and this makes them distinct from other chemotherapy drugs. Monoclonal antibodies are manufactured by exposing a viral component to white blood cells and then the isolated proteins are mass produced by a cloning process in the laboratory [56]. Currently, two major categories of MAbs are being tested for COVID-19. The first category specifically target and neutralize the virus (casirivimab, imdevimab, bamlanivimab) and the second group of MAbs acts on the immunological system and decreases inflammatory conditions following infection (tocilizumab, sarilumab and siltuximab) [13].

Patents and patenting strategies for biotechnological products such as monoclonal antibodies are cumbersome. Most of the time, these complex biological molecules require more than one patent to cover all the aspects of the innovation [57]. Furthermore, the novelty in biotechnology originates mostly from a university/public supported/research institution. Patenting such technological advancements for commercial purposes or technology transfer requires several legal and procedural issues [58]. The available data indicated that till recently three classes of MAbs have received emergency use authorization. They are bamlanivimab, casirivimab-imdevimab combination and bamlanivimab-etesevimab combination [13].

Patent analysis suggested that MAbs tested for the treatment of COVID-19 are manufactured by recombinant DNA technology. The complexity in the manufacturing technique includes the production of crude protein through cell culture in a bioreactor, followed by a series of purification steps and finally the sterile filling methods. The process starts from the immunization of laboratory animals such as BALB/c [59]. This sensitizes the B lymphocytes against the antigen needed for the production of MAbs. Specific B cells are identified, fused and hybridized. Hybrid cells are cloned and their ability to produce the specific antibody is detected by ELISA. The successful identification of hybrid cells is the first major breakthrough that leads to mass-production and is done in special cell culture vessels [60]. Purification of the specific antibody is the next important step and requires different techniques such as ion-exchange chromatography and antigen-affinity chromatography. Finally, the purified antibodies need to be filled into vials under strict aseptic conditions [61].

A phylogenetic analysis revealed that SARS-CoV-2 has about 77% similarity with SARS-CoV-1. The glycosylated spike (S) proteins are considered the key components for infection and can be located on the surface of the virus. The components of ‘S’ protein can be differentiated into extracellular N-terminal, a transmembrane (TM) and short intracellular C-terminal segment [62]. The proteins present in them are divided as S1 and S2. S1 is comprised of an N-terminal, receptor binding domain (RBD) and is reported to be involved in receptor binding characteristics. On the other hand, S2 is made of a fusion peptide, TM domain, heptapeptide repeat sequence (HR-1 and HR-2) and a cytoplasmic domain. This subunit of ‘S’ protein is responsible for fusion of the viral component to the host cell membrane and also facilitates the release of the viral genome into the host cells [63].

MAbs have a neutralizing ability against several pathogens including SARS-CoV-2. They are reported to have potential for both a prevention as well as therapeutic purpose. After the identification of the pathway for COVID-19 infection, several attempts were made to produce interventions that target specific sites [64]. The virus was found to have special affinity to the angiotensin converting enzyme-2 (ACE-2). This site is utilized by the virus for binding and gaining entry into the host cells. Advancement in technology has revealed several details about the spike proteins including their atomic size and binding characteristics [65].

The majority of patents are registered for the treatment of SARS-CoV and some are also registered for diagnostic use [52]. The viral pathogenesis revealed that the receptor-binding domain (RBD) in the S protein of SARS-CoV is required not only for entry into host cells but also for the initiation of the host immune response [41]. Hence, most of the MAbs (>90%) are targeted against S proteins, including RBD. Since a cytokine storm is one of the complications of COVID-19, some of the MAbs are also targeted against proinflammatory mediators, such as TNF-α, IL-4, and IL-10 [66].

One of the complications of COVID-19 is reported to be a severe and serious inflammatory reaction. This has been linked to the sudden release of enormous amounts of cytokines (‘cytokine storm’), contributing in the development of a life-threatening inflammatory syndrome. Research is in progress to treat this hyperactive immunological condition by using MAbs [67]. The agents specifically target the cytokines responsible for inflammatory and proinflammatory processes such as interleukins (IL-6 and IL-8). Some of the drugs, such as tocilizumab, siltuximab and sarilumab, are being tested for this property. The data available from initial findings suggested that administration of these MAbs had reduced the mortality rate a significant amount in seriously ill COVID-19 patients [66,67,68].

Antibodies against glycoprotein enveloped pathogens such as SARS-CoV-2 contain two major antigen interacting sites: Fab (antigen-binding fragment) and Fc (crystallizable fragment). The Fab region of antibody binds to viral antigens and damages their binding ability, while the Fc region activates antiviral effector cells leading to antibody-dependent toxicity to the virus [69]. However, sometimes, especially if the binding to glycoprotein is altered, it might then contribute in antibody-dependent enhancement (ADE) of virus uptake by macrophages, resulting in hyper-inflammatory responses. This condition is reported to contribute in the complications of immunotherapy [70].

Some modifications have been described in the production of MAbs from established techniques. In one such method, transgenic H_2_L_2_ mice were used that specifically code for chimeric immunoglobulins having human variable heavy and light chains. The neutralizing antibodies were isolated from the plasma. The identity of the antibodies was established by ELISA-cross reactivity with the SARS-S_1_ subunit. Then the chimeric antibodies were modified into fully human immunoglobulins by duplicating the human variable heavy and light chain regions in the human IgG1 isotope backbone [69]. Research has indicated that the SARS2-S_1B_ subunit consists of a core domain and a receptor binding domain which hangs out, and this directly allows interaction with the host receptor [71]. Potent neutralizing monoclonal antibodies are reported to target this site and prevent the progression of infection. The combined use of two or more monoclonal antibodies is being tested since they can produce multiple actions at different binding sites between the virus and host cell and could potentially produce a synergistic inhibitory action [72]. Studies are also being undertaken to modify the Fc region of the antibody so that the half-life of MAbs could be extended [73].

Being protein molecules, the action of MAbs in the body is not going to be straightforward. They might play multiple roles and activate several other functions in the host system, leading to unwanted and undesirable effects [73]. Therefore, adverse reactions after the administration of MAbs are also unpredictable in many individuals. The common adverse events reported were hypersensitive reactions, anaphylaxis, nausea, dizziness, pruritis and diarrhea. These reactions must be carefully monitored and there must be a plan to treat them in advance [74]. In addition to this, other major drawbacks of MAbs are the unpredictable bioavailability profiles. Studies suggest that the serum concentration of MAbs in several organs is not uniformly distributed and one such organ is the lung. Being the major site of COVID-19, uncertainty in the serum levels of MAbs in lungs needs to be assessed thoroughly before establishing the efficacy of the compounds [75].

The occurrence of variants of SARS-CoV-2 is the next major limitation of MAbs, since these strains have shown resistance to several drugs. There has been concern regarding the escape mechanisms adopted by SARS-CoV-2 variants against the actions of MAbs. A preclinical study suggested that some of the mutated strains of SARS-CoV-2 have modified structures of ‘S’ protein epitopes [76]. The findings indicated that the alteration in the key components such as epitopes might contribute to the development of resistance to most of the preventive as well as therapeutic interventions, including MAbs. Further, the RNA-virus in the past has displayed higher rates of mutation. In this context, efforts are being made to target different sites of the virus by using a combination of drugs [77]. The following sections briefly summarize the available information for some of the important patented SARS-CoV-2 monoclonal antibodies. Regeneron has patented its two innovative monoclonal antibodies (REGN10933, Casirivimab; and REGN10987, Imdevimab). The description claims that the agents are high-affinity IgG1 antibodies that specifically bind to RBD S proteins. REGN10933 was isolated from a VelocImmune human antibody mouse, while REGN10987 was from the B cells of COVID-19 recovered patients. The two monoclonal antibodies are recommended to be administered together to achieve higher efficacy and must not be given as a monotherapy [78].

The combined therapy of the two monoclonal antibodies, bind at distinct regions of RBD, thus overlapping the binding sites of the virus for ACE2. Further, the individual bindings to RBD were found to be non-competitive and non-overlapping. The available data suggests that the cocktail therapy of MAbs was effective for most of the circulating variants of COVID-19. Common adverse reactions were reported to be headache, dyspnea, nausea, chills, chest pain and pyrexia. In addition, exacerbation of viral infection due to antibody-dependent enhancement is also suspected [79].

The description mentioned in W020050588815 indicates bispecific human antibodies for the management of inflammatory disorders, autoimmune diseases, neurodegenerative disease, bacterial and viral infections. These antibodies have bispecific properties and immune-conjugates with a high specificity to IP-10 for their action [80]. Patent information W020117095875 describes the production of human antibodies and their binding characteristics specifically to IP-10 cytokines. The description also claims that this novel agent suppresses the inflammatory action in Cynomolgus macaques within 3 days at a 0.5 mg/kg dose [54,81].

The patent application W0200505824 describes the production of humanized anti-DC-SIGN antibodies. DC-SIGN/CD209 is the type of transmembrane adhesion molecule expressed mainly in lung alveolar macrophages and interstitial dendritic cells. The patent information suggests that the antibodies inhibited viral binding, infection, and transmission of viruses, including SARS-CoV [54,82].

In another patent application, monoclonal antibodies were synthesized from recovered COVID-19 patients and were used for both the diagnosis and treatment of SARS. The nucleic acid molecules can be delivered through vectors for prevention of the disease [57,83]. A Chinese patent description indicates the application of immunoglobulins such as polyclonal and monoclonal antibodies for the diagnosis, prevention, and treatment of SARS [84,85]. Another Chinese patent application reports the use of newly designed and developed monoclonal antibodies against the S protein receptor binding domain. These antibodies were claimed to be used for both the diagnosis and treatment of COVID-19, mostly in emergencies, and for analyzing the immunogenesis of spike proteins [85,86].

Table 4 lists other significant patent applications filed this year (2021) in the World Intellectual Property Organization (WIPO). The organization primarily aids in the pursuit of international patent protection and facilitates the decision to grant a patent.

Further, the discovery of S protein subunits, 3CPro, 3CLPro, and PLPro are extremely important targets that require extensive research. Monoclonal antibodies are highly specific in their targets and must be designed and developed to attack these targets [87]. In the event of an emergency with several variants of SARS-CoV-2, it is also essential to test the combination of monoclonal antibodies with other agents such as biological amines, antiviral and immunomodulatory drugs; these must be researched for safety and efficacy in the treatment of SARS-CoV-2.

## 6. Conclusions

The present study reviewed technological innovation in the development of monoclonal antibodies. Advanced research techniques and sophisticated instruments have guided us in understanding the pathways of SARS-CoV-2 infection as well as the key enzymes and proteins necessary for viral replication. Highly specific in their target, monoclonal antibodies are designed and developed to attack one or more sites of viral pathogenesis. The majority of the registered patents for monoclonal antibodies target the S protein and its binding sites. Considering the current situation where SARS-CoV-2 is being mutated at very regular intervals, more aggressive approaches, such as the combination of monoclonal antibodies with other known therapeutic interventions, need to be evaluated clinically to prevent COVID-19 spread and defeat the pandemic.

## Figures and Tables

**Table 1:** List of important drugs in the pipeline against COVID-19 [2,8].

Sl No.	Class	Target/Mechanism	Examples
1	Protease inhibitors	3C and 3C-Like protease (3C^Pro^, 3CL^Pro^), Popain-like protease (PL^Pro^)	N-butyl-benzimidazolylamino-toluene derivatives, Phytochemicals, such as scutellarin, quercetagetin, myricetin and robinetin.
2	Non-structural proteins inhibitors	Helicase	Aryl diketoacids
3	Peptides	Non-antigenic polymers to enhance immunity	Thymosin α1 peptide
4	RNA products	Down-regulates host ACE2 receptor level	Soluble ACE2 in DNA encoding form
		SARS-mRNA	Robozyme (an antisense RNA)
		SARS M protein expression	_si_RNA-M1 (Double stranded RNA)
5	Vaccines	S protein	Vector-based and attenuated vaccines by intra-nasal route
6	Inhibitors of unknown target	Disruption of viral protein-cellular interaction	Amiodarone, Dronedarone, mono-desethyl-amiodarone

Note: ACE-2—Angiotensin converting enzyme-2, SARS—Severe Acute Respiratory Syndrome, S protein—Spike protein.

**Table 2:** Chronology of technical innovations in the development of monoclonal antibodies [28,29].

Sl No.	Technique	Technical Design	Examples	Target	Approved Year
1	Hybridoma	Murine	Muromonab	CD3	1986
		Chimeric	Abciximab	GP IIb/IIIa	1994
		Humanized	Palivizumab	RSV	1998
2	Phage display	Human	Adalimumab	TNF-α	2002
		Murine	Moxetumomab	CD22	2018
3	Transgenic mice	Human (XenoMouse)	Panitumumab	EGFR	2006
		Human (HuMabMouse)	Ustekinumab	IL-12	2009
		Human (Veloclmmune Mouse)	Alirocumab	PCSK9	2015

Note: CD—cluster of differentiation; GP—glycoprotein; RSV—respiratory syncytial virus; TNF—tumor necrosis factor; EGFR—epidermal growth factor receptor; IL—Interleukins; PCSK9—Proprotein convertase subtilisin/kexin type 9.

**Table 3:** Important MAb patents registered for treating COVID-19 [54].

Sl No.	Patent Number	Description	Target Antigen	Organization
1	WO2009128963	Method of preparation and use of human monoclonal antibodies for neutralizing the action of SARS-CoV.	Spike protein	Institute for Research in Biomedicine
2	WO2007044695	Information about monoclonal antibodies used for diagnosis and treatment of SARS-coronavirus-associated disease and evaluating the efficacy of vaccine or anti-SARS agent.	Spike protein	Dana-Farber Cancer Institute
3	CN1911963	Technique of production and use of a monoclonal antibody against severe acute respiratory syndrome coronavirus.	RBD of S protein	Chinese Academy of Sciences
4	WO2006095180	Human monoclonal antibodies to treat the infection in patients caused by SARS-associated coronavirus.	S2 protein	Ultra Biotech Ltd.; University of California
5	WO2006086561	Production and therapeutic application of neutralizing monoclonal antibodies against severe acute respiratory syndrome-associated coronavirus.	Spike protein	New York Blood Center, Inc.
6	WO2005007671	Production of monoclonal antibodies against the peptides derived from SARS virus E2, N-terminal-alpha helix or C-terminal-alpha helix of virus.	Spike protein	Epitomics, Inc
7	CN1673231	Synthesis of a monoclonal antibody targeted against N proteins of SARS coronavirus and testing its clinical use in the treatment of SARS infections.	Spike protein	Chinese Academy of Sciences
8	US20060240551	Production and clinical evaluation of monoclonal antibodies that neutralize the pathogenesis of severe acute respiratory syndrome-associated Coronavirus.	Spike protein	New York Blood Center, Inc.
9	WO2005054469	Production of anti-SARS-coronavirus monoclonal antibodies for diagnosis and treatment and for testing its use in vaccine preparation.	Spike protein	Health Canada
10	US20050069869	New human monoclonal antibodies against spike (S) proteins of SARS and testing their diagnostic and therapeutic application.	Spike protein	University of Massachusetts
11	CN1566155	Library-driven production of human monoclonal antibodies against SARS virus caused infection.	S, N, and M Proteins	Igcon Therapeutics Co., Ltd.; Genetastix
12	CN1660912	Production and testing the use of a new class of monoclonal antibodies against human interleukin.	Il-8	Ye Qingwei

Note: RBD—Receptor binding domains, Il—Interleukins, S, N, M proteins—Spike, Nucleocapsid, Membrane proteins.

**Table 4:** List of important patent applications currently filed (2021) for MAbs against COVID-19 in WIPO [55].

Patent Number	Patent Information	Target Antigen	Principle Investigators
WO 2021158521 A1 20210812	Neutralizing monoclonal antibody variants targeting SARS CoV-2 for use in diagnosis, prophylaxis, and treatment of SARS CoV-2 infection.	Spike protein and/or its receptor binding domain	Davide C, Katja F, Martina B, et al.
CN 113004395 A 20210622	Production of monoclonal antibody against SARS-CoV-2 and application thereof in immunoassay of SARS CoV-2.	NP protein	Li Z, Xingsu G, Binyang Z, et al.
CN 112940110 A 20210611	Production of anti-SARS-CoV-2 N protein monoclonal antibodies for diagnosis and treatment of COVID-19.	N protein	Yaoqing C, Bing H, Shuning L, et al.
CN 112794899 A 20210514	Human anti-SARS-CoV-2 neutralizing monoclonal antibodies for diagnosis, prevention, and treatment of COVID-19.	Viral receptor binding domain (RBD)	Lei C, Tengsen G, Min D, et al.
CN 112724248 A 20210430	Humanized anti-SARS-CoV-2 spike protein nanobodies for diagnosis, prevention, and treatment of COVID-19.	Receptor binding domain	Xilin W, Zhiwei W.
CN 112661841 A 20210416	Anti-SARS-CoV-2 S2 protein human monoclonal antibody 17-2 for combination therapy with S1-RBD/S1-NTD epitope-neutralizing antibody and for prevention and treatment of COVID-19.	Epitope S1-RBD and S1-NTD	Lei Y, Yingfen W, Wenjing G, et al.
CN 112625136 A 20210409	Bi-specific antibody having neutralizing activity against two epitopes of SARS-CoV-2 spike protein for diagnosis, prevention, and treatment of COVID-19.	Two epitopes of SARS-CoV-2 spike protein	Guojun L, Chanjuan L, Junbin S, et al.
CN 112574300 A 20210330	Human anti-SARS-CoV-2 S protein monoclonal antibody for diagnosis, prevention, and treatment of SAR-COV-2 infection.	Spike protein	Xiaochun W, and Junxin L.
CN 112521496 A 20210319	Anti-SARS-CoV-2 spike protein RBD domain monoclonal antibodies for diagnosis and treatment of COVID-19.	Spike protein RBD domain	Ke D, Zhaowei G, Xi W, et al.
CN 112409488 A 20210226	Preparation of monoclonal anti-human ACE2 antibody for ACE2 detection, prevention, or treatment of various coronavirus-related disease.	Human ACE2	Chunhe W, Yuning C, Yili C, et al.
CN 112225806 A 20210115	Preparation of human antibodies specific to SARS-CoV-2 spike RBD protein for diagnosis and therapy of SARS-CoV-2 infection, SARS, COVID-19 or related disease.	Spike RBD protein	Yafeng L.
CN 112210004 A 20210112	Preparation of monoclonal anti-SARS-CoV-2 spike protein antibodies for diagnosis and treatment of COVID-19.	Spike protein	Yang W, Xuefeng N, Chunlin W, et al.
CN 112175073 A 20210105	Preparation of broad spectrum neutralizing anti-SARS-CoV-2 spike protein antibodies for diagnosis, prevention, and treatment of COVID-19.	Spike protein	Jinghe H, Fan W, Mei L, et al.
CN 112175071 A 20210105	Preparation of novel anti-SARS-CoV-2 spike protein monoclonal antibodies for treatment of COVID-19.	Spike protein	Jingui Y, Lei Z, Lianjun M, et al.
CN 112159469 A 20210101	anti-SARS-CoV-2 S1-RBD antibodies derived from in vitro monoclonal B cells and high throughput screening for treatment and/or prevention of COVID-19.	Spike S1-RBD	Jinghe H, Fan W, Mei L, et al.

Note: SARS CoV-2—severe acute respiratory syndrome coronavirus-2; RBD—Receptor binding domain; S, N, proteins—Spike, Nucleocapsid, proteins; ACE2—Angiotensin converting enzyme-2.

## Abbreviations

COVID-19	Coronavirus disease 2019
SARS-CoV-2	Severe Acute Respiratory Syndrome Coronavirus-2
MAbs	Monoclonal antibodies
MERS-CoV	Middle East Respiratory Syndrome Coronavirus
RT-PCR	Reverse transcriptase polymerase chain reaction
RNA	Ribonucleic acid
ACE-2	Angiotensin converting enzyme-2
S protein	Spike protein
S1 and S2	Subunits of Spike protein
C^Pro^	Chymotrypsin-like protease
CL^Pro^	3C-like protease
PL^Pro^	Papain-like protease
DNA	Deoxy ribonucleic acid
WIPO	World Intellectual Property Organization
FDA	Food and drug administration
CD-3	Cluster of differentiation-3
GP	Glycoprotein
RSV	Respiratory syncytial virus
TNF	Tumor necrosis factor
EGFR	Epidermal growth factor receptor
IL	Interleukins
PCSK9	Pro-protein convertase subtilisin /kexin type-9
Fab	Fragment antigen binding
SCFv	Single chain variable fragment
SdAb	Single domain antibody
Fc	Fragment crystallisable
RBD	Receptor binding domain
FGF-23	Fibroblast growth factor-23
SNM protein	Spike, nucleocapsid, membrane protein
BALB/c	Bagg Albino strain
ELISA	Enzyme linked immunosorbent assay
HR-1/HR-2	Heptapeptide repeat sequence-1/2
H_2_L_2_ transgenic mice	Two heavy chain and two light chain transgenic mice
IgG	Immunoglobulin G
